# SAUTE: sequence assembly using target enrichment

**DOI:** 10.1186/s12859-021-04174-9

**Published:** 2021-07-21

**Authors:** Alexandre Souvorov, Richa Agarwala

**Affiliations:** NCBI/NLM/NIH/DHHS, 8600 Rockville Pike, Bethesda, MD 20894 USA

**Keywords:** Illumina reads, De-novo assembly, de Bruijn graphs, Antimicrobial resistance, RNA-seq

## Abstract

**Background:**

Illumina is the dominant sequencing technology at this time. Short length, short insert size, some systematic biases, and low-level carryover contamination in Illumina reads continue to make assembly of repeated regions a challenging problem. Some applications also require finding multiple well supported variants for assembled regions.

**Results:**

To facilitate assembly of repeat regions and to report multiple well supported variants when a user can provide target sequences to assist the assembly, we propose SAUTE and SAUTE_PROT assemblers. Both assemblers use de Bruijn graph on reads. Targets can be transcripts or proteins for RNA-seq reads and transcripts, proteins, or genomic regions for genomic reads. Target sequences are nucleotide and protein sequences for SAUTE and SAUTE_PROT, respectively.

**Conclusions:**

For RNA-seq, comparisons with Trinity, rnaSPAdes, SPAligner, and SPAdes assembly of reads aligned to target proteins by DIAMOND show that SAUTE_PROT finds more coding sequences that translate to benchmark proteins. Using AMRFinderPlus calls, we find SAUTE has higher sensitivity and precision than SPAdes, plasmidSPAdes, SPAligner, and SPAdes assembly of reads aligned to target regions by HISAT2. It also has better sensitivity than SKESA but worse precision.

**Supplementary Information:**

The online version contains supplementary material available at 10.1186/s12859-021-04174-9.

## Background

Long read sequencing technologies such as PacBio and Nanopore are becoming increasingly popular. However, Illumina’s short read approach continues to be the dominant sequencing technology at this time. Illumina reads have very low insertion deletion error rate but suffer from short length, short insert size, some systematic biases, and low-level carryover contamination from earlier runs [1–3]. These shortcomings in Illumina reads continue to make assembly of repeated regions a challenging problem [4–10].

The two basic strategies for assembly are reference based methods that align reads to a reference genome [11–16] and de-novo methods that use the overlaps among the reads themselves for assembly, without the need for a reference sequence [17–22]. Tools that align reads to genome graphs, such as, GraphAligner [23] and SPAligner[24], are emerging and can also be used for producing assemblies. In this manuscript, we present two tools: SAUTE (Sequence Assembly Using Target Enrichment) and SAUTE_PROT. Both assemblers take advantage of user provided *target sequences* to limit the search and heuristics to find homologs of target sequences supported by reads. We use “target” instead of “reference” for user provided sequences as the term reference typically refers to reference genomes used by reference based assembly methods.

Target sequences are nucleotide and protein sequences for SAUTE and SAUTE_PROT, respectively. Conceptually, both tools build a de Bruijn graph on reads, search for subgraphs in the de Bruijn graph that have k-mers with a sufficiently high score to k-mers in the target sequence, and extend paths in subgraphs to find long enough assemblies that maintain a sufficiently high alignment score to a target sequence. We note that our approach is different from reference based assembly methods for RNA-seq reads as we are not aligning reads to a reference genome or explicitly generating splice variants by searching for splicing events. It is also different from tools aligning reads to genome graphs as we build the graph on reads.

Target sequences can be transcripts or proteins for RNA-seq reads and transcripts, proteins, or genomic regions for genomic reads. Target sequences can be from the same species as the species of the sample reads were generated from or a different species. However, as the type of target sequences SAUTE and SAUTE_PROT receive is different, the scoring schemes for aligning subsequences of a target sequence to k-mers or paths in the de Bruijn graph constructed from reads differ between the two tools. Additionally, k-mer size for SAUTE_PROT needs to be a multiple of three. Heuristics are designed to reduce the effect of strand specific errors in Illumina sequencing on the quality of the assembly. We show the advantages of this approach for genomic and RNA-seq assembly in two scenarios where the researcher has nucleotide or protein target sequences: assembling coding sequences using distant orthologs and reporting multiple well supported variants for assembled regions. In the rest of the manuscript, we use SAUTE to mean both SAUTE and SAUTE_PROT unless specified otherwise.

Assembling RNA-seq reads using proteins of distant orthologs is of interest as we have reference genome assemblies for a limited number of species and RNA-seq reads from a much larger number of species. For example, as of October 2020, there were 89 Drosophila species with RNA-seq reads sequenced using Illumina in Sequence Read Archive (SRA) of which 42 species had any assembly at NCBI and only 18 species had an assembled genome with N50 of at least one megabase.

Assembling and reporting multiple well supported variants for an assembly is important for several applications, such as, finding antimicrobial resistance (AMR) and virulence genes in microbial genomes that is a serious global threat [25–28], annotating genome features that can impact clinical genome diagnostics [29, 30], and finding mutations in cancers for personalized medicine [31–33].

In order to assess the assembly approach in SAUTE, we assembled (i) RNA-seq reads for five species using orthologous pairs in Benchmarking Universal Single-Copy Orthologs (BUSCO) [34] for defining target and benchmark proteins, (ii) RNA-seq reads for five read sets from BioProject PRJNA590287 using *Drosophila innubila* and *Drosophila melanogaster* proteins as targets and *Drosophila melanogaster* proteins as benchmark, and (iii) genomic reads from 763 microbial read sets from FDA-ARGOS [35] benchmark set using virulence and antimicrobial resistance genes as target sequences.

A coding sequence assembled by a method is said to recover a benchmark protein *perfectly* if it finds the full length of the protein at 100% identity. A coding sequence assembled by a method is said to recover a benchmark protein as *essentially complete* if at least 90% of the protein is recovered at $\ge$ 97% identity. We show that when percent identity of the alignment between read and target proteins is at least 75%, SAUTE finds more coding sequences that recover benchmark proteins perfectly or as essentially complete compared to de-novo assemblers rnaSPAdes and Trinity, assemblies reported by graph aligner SPAligner on assembly graphs generated by rnaSPAdes, and assemblies generated for each target sequence *T* by SPAdes on subsets of reads that align to *T* by DIAMOND[36]. When identity is below 75%, de-novo assembly methods occasionally outperform SAUTE.

Using AMRFinderPlus[37, 38] calls on reference genomes as benchmark, we find that SAUTE has higher sensitivity and precision compared to calls made on genome assemblies by SPAdes[39], calls made on genome assemblies by SPAdes supplemented by calls made on plasmidSPAdes[40] assemblies, calls made on assemblies generated by SPAligner on assembly graphs generated by SPAdes, and calls made on assemblies generated for each target sequence *T* by SPAdes on subsets of reads that align to *T* by HISAT2 [41]. It also has better sensitivity compared to calls made on genome assemblies by SKESA[42] but worse precision.

SAUTE can access reads directly from SRA and from files. SAUTE is currently used in production at NCBI for assembling AMR genes for the pathogen detection project [43, 44]. Software for SAUTE is freely available [45] (see Availability and requirements).

Default values of the parameters for SAUTE are expected to give good results but for read sets that have low-coverage, we change two coverage parameters, minimum k-mer count and number of aligned reads for filtering, to one instead of the default of two. Runs with defaults and with low coverage parameters are referred to as SAUTE default and SAUTE low, respectively.

## Implementation

We present the algorithm design for SAUTE, some dependencies, important optimization details, and command lines used for doing the runs.

### Algorithm design for SAUTE

The main steps for assembling reads using a target sequence in SAUTE are as follows:

- **Read input and trim reads:** SAUTE can receive input from files (fasta or fastq) or directly from SRA.
- **Build two de Bruijn graphs (DBG):** By default, SAUTE automatically chooses two k-mer sizes for building DBG. The longer *primary* k-mer is chosen as the largest odd integer that is at most half of the length of reads. The shorter *secondary* k-mer is chosen as larger of 21 and the largest odd integer that is at most a fifth of the length of reads. If target has protein sequences, then an additional condition of k-mer sizes being a multiple of three is enforced. A user can explicitly choose to provide both k-mer sizes and override the default behavior. If primary k-mer computed is shorter than 21, automatic k-mer size detection fails and the user is asked to provide the k-mer sizes to use. DBG for primary and secondary k-mers are referred to as primary DBG and secondary DBG, respectively. We use *read count* for k-mer *K* to refer to the number of times *K* is present in reads.
- **Find and assemble subgraphs:** For each target sequence, find subgraphs in the primary DBG using *seeds* (described below). Assemble by extending paths. When a path cannot be extended using primary DBG, or has only one choice for extension and certain read count conditions are met (described later), check if secondary DBG can be used to extend the path at this location while reverting to primary k-mer for the next step of the assembly. The graphs produced after all extensions using primary and secondary DBG are called *assembled graphs* generated using the target. For aligning a path to target, a dynamic programming algorithm is used. SAUTE uses match *reward*, mismatch *penalty*, gap open, and gap extend parameters. SAUTE_PROT translates the assembled path on the fly using the genetic code specified by the user, and uses BLOSUM62 substitution matrix, gap open, and gap extend penalties. If the parameter that allows frameshifts in the assembly is used, a larger gap open penalty is used for such gaps.
- **Filter graphs:** As k-mers have less information than reads, some paths in an assembled graph are not supported by reads. We filter such connections in the assembled graph using alignments of reads and read pairs to the assembled graph.
- **Extend ends of paths:** When a user wishes to assemble additional sequence beyond the ends of what can be assembled using the target, a flag called extend_ends can be provided. In this case, each end of each path in the assembled graph that remains after filtering is extended using the primary DBG as long as no alternate choice is available for any base in the extension.
- **Report output:** The result is reported in graphical fragment assembly (GFA) [46] output format and two fasta files.

Next, we describe selection of seeds for finding subgraphs, process for assembling paths, and filtering by reads and read pairs when assembling using a target sequence. If more than one target sequence is present in the target set, additional steps taken to remove redundancy are discussed. Finally, we present how assemblies are reported in three output files.

#### Seed k-mers

Let *k* be the size of the primary k-mer, *S* a k-mer in primary DBG, and *p* a position in the target sequence.

If target is a nucleotide sequence, let *R* be the sequence in the target starting at position *p* of length *k* and *M* be the number of matches in the hamming distance between *R* and *S*. *S* is said to have a *good alignment* at position *p* in the target sequence if it has following properties:

- A short suffix of size *w* (parameter word (default 8 bp)) at the end of *S* is an exact match to the last *w* bases of *R*.
- *M* is greater than minimum of $k-1$ and *V* computed as $V = \lfloor k/10 \rfloor + k * penalty / (reward + penalty)$

If target sequence is a protein sequence, let *R* be the sequence in target starting at position *p* of length *k*/3, *T* be the translation of *S*, $R_{self}$ be the self-score of *R* aligning to itself using BLOSUM62, and $R_{align}$ be the score of *R* aligned to *T* without gaps using BLOSUM62. *S* is said to have a *good alignment* at position *p* in target if it has following properties:

- A short suffix of size *w* (parameter word (default 12 bp translated to 4 aa)) in *S* translated to *w*/3 residues at the end of *T* is an exact match to the last *w*/3 residues of *R*.
- $$R_{align} > 0.75 * R_{self}$$

For comparing reverse complement of *S* to the target, the same rules for scores apply but instead of the last *w* bases, the first *w* bases of the reverse complement of *S* or *w*/3 residues of translation of the reverse complement of *S* are matched to the first *w* bases or *w*/3 residues of *R*. This is because a hash table is used for finding exact matches and the hash is created using the last *w* bases of k-mers.

Let *L* be the read count for k-mer *K*. *K* is called a *seed k-mer* if it has following properties:

- *K* is in primary DBG.
- *K* has a good alignment to exactly one position *p* in the target sequence.
- *K* has at least one extension of 100 bp from both ends of *K* in the primary DBG. However, if protect_reference_ends flag is used, then this condition is not checked in the direction where *p* is less than 100 bp or 34 aa from the end of the target sequence.
- $$L > 1$$
- Let *N* be the maximum read count for any k-mer satisfying above four conditions at the same target position *p*. Then, $L \ge N * \mathtt{fraction}$ (default 0.05)
- Let *X* be the number of different k-mers at position p satisfying the above conditions. If $X \ge \mathtt{kmer\_complexity}$ (default 2000), then all seed k-mers for position *p* are deleted.

Seed k-mers serve as starting points for assembly. The last condition above affects positions in highly repetitive intervals. The k-mer intervals starting at all such positions are hard masked by replacing the target sequence by Ns for nucleotides or by Xs for proteins. This effectively removes such intervals from the alignment process and will likely result in fragmented assembly.

#### Assembling and aligning paths

Seed k-mers are extended to the left from the position of the target sequence preceding the beginning of the seed and to the right after the end of the seed, referred to as left extension and right extension, respectively. The extensions do not include the seed k-mer, are independent of each other, and follow exactly the same procedure. We describe right extension below and use *path* to refer to one of the several possible right extensions. Same procedure easily extends for left extension.

At any stage, extension attempt results in three possibilities: (i) no k-mer extension is possible, (ii) only one k-mer extension is possible, or (iii) there are alternate choices. In the first case, the end of the path (commonly called as dead-end) has been reached and no further extension is possible.

In the third case, all k-mer choices using this k-mer length with counts below the threshold for extension fraction with respect to the maximum count for any choice are considered as noise and dropped. If more than one choice for extension survives this count based filtering, potential Illumina strand specific systematic error signatures are evaluated. The program does this by comparing counts observed on both strands. If there is a choice with counts balanced on both strands, all choices with counts seen in predominately one strand are dropped. If more than one choice for extension survives this strand based filtering, the position is called a *fork* position and each choice is preserved as a valid branch for future extensions. The branch with the maximum count is explored next using the same process as if there was only one k-mer extension. If more than one branch has the same maximum count, ties are broken lexicographically using the sequence of the branch.

In the second case, there is no branching and we assess alignment quality of the path with the extension to see if the path meets the criteria for being similar to the target sequence. The initial state before the first extension is that *best scoring position* is unknown and *best score* is 0. The alignment score of the path extended by the base is compared to the current best score for this path. The comparison results in three possibilities: (a) if the score is better, then the best score and the best scoring position are updated and extension along the same path is continued, (b) if the current score is below the best score by at most the drop-off value, extension along the same path is continued, and (c) if the current score is below the best score by more than the drop-off value, the path and all stored branch points are clipped to the best scoring position. Such positions are marked as positions where forks existed and are positions to be used for filtering with reads and read pairs. The program continues with the next stored branch or the next seed k-mer.

A path is extended by one base only if the extension from the new k-mer produced by addition of the base to the previous k-mer (last k-mer of the end of the path) is also possible using the same criteria used for extending from previous k-mer to the new k-mer. If at the end of all extensions, the best scoring position is still unknown, then no right extension was possible. Otherwise, every k-mer in extension found that is also a seed k-mer is removed from the list of remaining seed k-mers.

Secondary DBG is used to find additional paths in regions suspected to have low coverage. It comes in play in the first two cases mentioned above. In the first case when no base is present in the primary DBG for extension, we consider extension using secondary DBG. In the second case where only one extension is possible in the primary DBG, if the read count for the k-mer with added base is at most secondary k-mer threshold (default 1), then secondary DBG is also considered for extension. We note that with the default minimum count of 2 and secondary k-mer threshold of 1, the read count condition is not met in the second case as every k-mer in primary DBG has count at least 2. The condition can be met only when secondary k-mer threshold is at least as large as minimum count, such as, with SAUTE low option where minimum count is set to 1.

#### Filtering by reads and pairs

For paired reads, SAUTE computes the insert size using de-novo connections of the mates as described in SKESA[42]. Three additional parameters needed for removing erroneous paths in assembled graph are number of reads needed to *confirm* a fork called aligned_count (default 2), number of reads that *contradict* a fork called not_aligned_count (default 3), and length of portion not aligned to the path not_aligned_len (default 10). We will use *Y* for half of not_aligned_len.

**Fig. 1:**
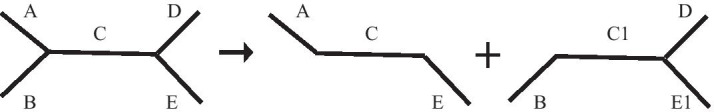
Forks for filtering by reads and read pairs

The paths that can be checked by reads have the graph structure shown in Fig. 1. A segment C is connected to two forks on either end of the segment. The number of choices at each fork is at least two. Figure 1 shows that the left fork has segments A and B and the right fork has segments D and E. These forks may be present in the assembled graph or marked as positions with a fork during the assembly process. For each of the four paths in Fig. 1, namely A-C-D, A-C-E, B-C-D, and B-C-E, we compute confirm and contradict counts. Below we describe how to check path A-C-D; same method easily extends to any other path.

Find reads that have a substring with same sequence as that for segment C. For each such read *R*, align the substring in *R* to C and extend it on both ends on path A-C-D without mismatches. If the alignment has at least *Y* bases aligned to A and *Y* bases aligned to D, then *R* is said to confirm A-C-D path. However, if the condition is satisfied for A [D] but no base of D [A] is in the alignment and there are at least $2*Y$ bases in the unaligned portion available for matching to D [A], then we say that the read contradicts A-C-D path. The path is considered incorrect if the number of reads contradicting the path reaches threshold not_aligned_count and the number of reads confirming the path is below aligned_count. In this case the path is removed and often results in duplication of some segments in the assembled graph as shown by E and E1 in Fig. 1.

For checking by pairs, we have one mate aligning to fork on the left and the other mate aligning to fork on the right with distance between the forks in the range as computed earlier for the insert size and in the expected orientation. Alignments for confirming a fork, contradicting a fork, and formula used is same as for reads above except that one fork is checked by one mate and the other fork is checked by the other mate.

#### Multiple sequences in target set

If input target has more than one sequence, flag keep_subgraphs controls whether each sequence is assembled independently or if some are removed as redundant. If this flag is used, then each target sequence is assembled independently. Otherwise, assembled graphs are checked for redundancy before and after the filtering step. For an assembled graph *X* to be removed, there must exist another assembled graph *G* such that all k-mers in *X* are also present in *G* where *k* is size of secondary k-mer. Removing such graph reduces the running time without affecting results in almost all cases. The exception is when the target set contains sequences for the fused genes in addition to the individual genes that were fused. In this case, only the assembled graph for the fused gene would survive the redundancy removal and graphs for individual genes would be removed as redundant.

#### Output

**Fig. 2:**
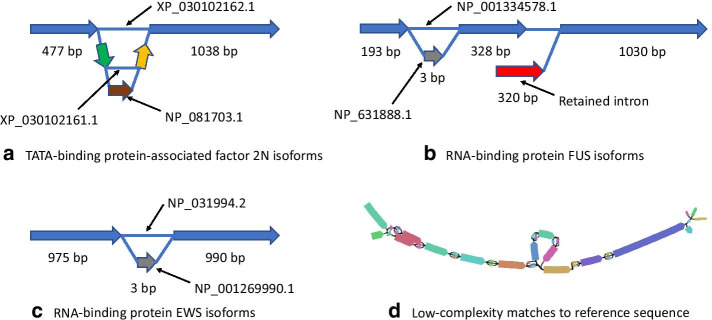
Four subgraphs resulting from assembly of mouse reads using NP_631888.1 target sequence by SAUTE low are shown as graphs A, B, C, and D. Graph D consists of k-mers from low-complexity region of the target

The assembled graph is reported in GFA format. Key features of this format are segments (nodes) and links (edges). Segments specify the name for segment and its sequence. GFA format graphs can be viewed using Bandage [47] that was used for producing part D of Fig. 2.

Reporting of assemblies produced is controlled by target_coverage and min_hit_len. The default for target coverage is 0.5 so an assembly (before extending ends if extend_ends is specified) that has length at least half of the target sequence length will get reported. Instead, a user can specify desired minimum length for reporting an assembly using min_hit_len parameter. If total number of assemblies to be reported is at most max_variants (default 1000), then all assemblies in fasta format are saved in the output file specified by all_variants. Otherwise, 1000 distinct assemblies are reported where for each assembly we compute sum of read counts for all k-mers of size secondary k-mer in the assembly, sort assemblies using this count in descending order, and report top 1000.

In cases where the total number of assemblies is extremely large, it may be useful to report best supported path for each link in the graph. For this limited usage, we report all such sequences in fasta format that are saved in the output file specified by selected_variants. When more than one link produces the same sequence, it is reported only once.

### Dependencies

SAUTE shares some modules with SKESA. Some of these include reading input, trimming reads, computing insert size, k-mer counting method that has an option for using a hash table and a Bloom filter [48], k-mer search method, building DBG for a given k-mer size and a given minimum number of times the k-mer should be present in reads (called min_count). Additional code dependencies include (i) freely available Boost library [49], (ii) SRA toolkit library if direct access to SRA is desired for retrieving reads, and (iii) the long integer implementation from [50] for k-mers.

### Optimization

#### Optimization for extending same k-mer multiple times

A k-mer *K* can be a substring in several branches of an extension to the left or right of the same seed. If same extensions beyond *K* can be expected from more than one branch, it is useful to detect the situation and collapse the branches at *K*. To achieve this, we define *anchor* k-mers as follows.

Suppose a path *L* is extended by a base *b* resulting in last k-mer *A* in path *L* that includes *b*. Let the best scoring position in *L* be *p* and *d* the length of sequence between position *p* and base *b*. Then, tuple (*L*, *p*) is an anchor if and only if $d < k/4$.

When another path *Q* is adding base *b* that gives k-mer *A*, process checks whether the best scoring position for *A* in *Q* is also *p* or not. If it is, then *Q* merges with *L* at *A* and no further extensions are needed for *Q*. Otherwise, *Q* continues extension beyond *A* without merging with *L*.

#### Choices for assembling highly repetitive regions

Repetitive regions longer than the k-mer size could result in very complex output graph and excessive running time. To deal with this problem, SAUTE has the following heuristics:

- As explained in the section on seed k-mers, certain positions on the target may have a very large number of seed k-mers. Such positions are hard-masked and may lead to the graph being split in subgraphs; causing breaks in assembly.
- If extension enters a repetitive area, there can be a large number of positions where there are forks and the number of paths to follow may become very high. SAUTE calculates the average fork density for the suffix of size buf_length (default 200 bp) of the assembly. If the fork density is above the threshold specified by max_fork_density (default 0.1), assembling for the current seed k-mer is stopped and the program moves to the next seed k-mer.
- To perform filtering by reads and read pairs, SAUTE must expand the assembled graph at each fork by read length or insert length, respectively. For highly complex graph this may result in an astronomical number of sequences. If needed, SAUTE reduces the expansion length, so that the number of sequences is below the allowed threshold max_path (default 1000).
- Each successful step of reads and read pairs filtering removes a path from the assembled graph and reduces the number of sequences which could be generated from the graph. On the other hand, as explained earlier, it may increase the number of segments in the graph by duplicating some segments. In rare cases, it may result in an incomprehensible graph. If the number of segments increase more than 15 times the initial number, the filtering process is deemed unsuccessful, and the graph is returned to its original state.

### Commands for programs

Command lines for, say, running SRR4381672 for SKESA and SPAdes are as follows:

skesa –fastq SRR4381672_1.fq,SRR4381672_2.fq –cores 4 –memory 16 –use_paired_ends

spades.py -1 SRR4381672_1.fq -2 SRR4381672_2.fq -t 4 -m 16 -o spades.SRR4381672

For running plasmidSPAdes, option –plasmid is added to above command for SPAdes. Similarly, for running rnaSPAdes, option –rna is added to above command for SPAdes.

For SKESA, if direct SRA access is available, one can instead do the following:

skesa –sra_run SRR4381672 –cores 4 –memory 16 –use_paired_ends

For SAUTE default, the command line is as follows:

saute –reads SRR4381672_1.fq,SRR4381672_2.fq –targets target.fa –gfa SRR4381672.gfa –all_variants SRR4381672.vars.fa –selected_variants SRR4381672.sel.fa –extend_ends –keep_subgraphs –cores 100

For running SAUTE low, options

–min_count 1 –aligned_count 1

are added to above command line for SAUTE default.

For running SAUTE_PROT instead of SAUTE, saute is replaced by saute_prot in above command lines and parameter to specify –genetic_code to use for translation is added.

For running RNA-seq read sets, option

–protect_reference_ends

is added to SAUTE and SAUTE_PROT runs.

Command line for AMRFinderPlus run is as follows:

amrfinder –plus -n SRR4381672.vars.fa

Index for DIAMOND is built with command:

diamond makedb –in target.fa -d diamond_index.target

Reads for each mate are aligned to above index with command:

diamond blastx -d diamond_index.target -q SRR4381672_1.fq -o SRR4381672_1.tsv

Index for HISAT2 is built with command:

hisat2-build target.fa hisat2_index.target

Reads are aligned to above index with command:

hisat2 -x hisat2_index.target –no-head –no-spliced-alignment –no-sq -k 7000 -1 SRR4381672_1.fq -2 SRR4381672_2.fq

Command line for SPAligner is as follows:

spaligner spaligner_config.yaml -s target.fa -k 77 -g SRR4381672.gfa -t 8 $\texttt { -o SRR4381672.spaligner -d <option>}$

For nucleotide and protein target sequences, parameter -d has value pacbio and protein, respectively. The input graph specified with -g is the GFA files for assembly graph with scaffolds. Value of -k is determined manually by looking at the k-mer value in the last column of lines starting with L in the GFA file.

## Results and discussion

In this manuscript, we present analysis using three types of data sets [51]:

(i) *RNA-seq* BUSCO *set* that assembles one read set each for five species: human (*Homo sapiens*), mouse (*Mus musculus*), corn (*Zea mays*), worm (*Caenorhabditis elegans*), and thale cress (*Arabidopsis thaliana*). Orthologous protein pairs in BUSCO derived from OrthoDB v10.1 are used to define benchmark and target protein sequences.
(ii) *RNA-seq THO set* that assembles five randomly chosen read sets from PRJNA590287. This BioProject sequenced samples for *Drosophila melanogaster* to study effect of variations in genes in THO complex and piRNA pathway on transcription activity. We use *Drosophila melanogaster* and *Drosophila innubila* proteins for five gene families in THO gene complex as target sequences and *Drosophila melanogaster* proteins as benchmark.
(iii) *AMR set* that assembles genomic reads from 763 microbial read sets from FDA-ARGOS benchmark set. We use virulence and antimicrobial resistance sequences used by the pathogen detection pipeline as the target set.

We compare SAUTE to Trinity v2.9.1 and SPAdes v3.14.0, including rnaSPAdes, plasmidSPAdes, and SPAligner modules of SPAdes, as they are the most widely used assemblers. As the names imply, rnaSPAdes is for assembling RNA-seq reads, plasmidSPAdes is for assembling plasmids from whole genome sequencing data that may harbor some virulence and AMR genes, and SPAligner aligns reads to a genome graph. SPAligner in SPAdes v3.14.0 was used for nucleotide target sets but we had to use the publication version of SPAligner for protein target sets as authors are merging the code for protein target sets into release versions (personal communication). We also assembled AMR set with SKESA developed for assembling microbial read sets. AMRFinderPlus v3.8.4 was used for finding AMR gene calls. For the experiment of finding subset of reads that align to each target sequence and assembling those subsets using SPAdes, referred to as CLUSTER assemblies below, we used DIAMOND v2.0.7.145 for aligning reads to protein target sequences and HISAT2 v2.0.5 for aligning reads to nucleotide target sequences.

For assessment, alignment between protein sequences was done using BLASTP and between protein sequence and nucleotide assembly using TBLASTN of the BLAST suite of software. Defaults were used for both except that we turned low-complexity filtering and composition-based statistics off.

### Assessment of RNA-seq set using BUSCO protein pairs

Table 1 gives information for the RNA-seq BUSCO read sets used for each of the five species, the clade they belong to, and the number of species in OrthoDB for that clade after excluding *Ornithorhynchus anatinus* from the mammalian clade. We excluded *Ornithorhynchus anatinus* from our analysis as a large fraction of proteins for this species are shorter in OrthoDB than those in GenBank and also shorter than the corresponding orthologs for human and mouse (data not shown). This gives a total of 99 read and target set combinations. Assemblies were generated with SAUTE default, SAUTE low, rnaSPAdes, Trinity, SPAligner, and CLUSTER. Trinity failed to assemble thale cress and worm read sets. SPAligner assemblies were generated by aligning proteins in target sets on assembly graphs generated by rnaSPAdes. SAUTE low was run with three limits of 10, 100, and default of 1000 maximum variants (option max_variants) reported per graph.

Assessment criteria used proteins for the read species in the orthologous pairs defined in BUSCO for human, mouse, thale cress, and worm. As corn is not in OrthoDB, we used RefSeq proteins annotated on the corn reference assembly GCF_000005005.2 for finding orthologs for the proteins for 15 liliopsida species in OrthoDB. Additional file 1 lists all 99 combinations of read and target sets. For each combination, it also gives the median identity between orthologous protein pairs and number of benchmark proteins for the pair that are recovered perfectly or as essentially complete using coding regions assembled by each assembly method. For each of the five species, Table 2 displays subset of the data from Additional file 1. It shows the row with the smallest median identity, the row with smallest median identity that is at least 75%, and the row with the largest median identity. Also shown are rows for human reads assembled with mouse proteins and vice versa as the lowest median identity ortholog in mammalian clade for human and mouse is over 75% and these species pairs are of high interest.

**Table 1:** Read and target information for RNA-seq BUSCO set

Read sp.	SRA runs	Clade	Count
Corn	SRR1588569	liliopsida	15
Thale cress	SRR5344669, SRR5344670	eudicots	31
Worm	SRR10005501	nematoda	7
Mouse	SRR10982198	mammalia	23
Human	SRR1957703, SRR1957706	mammalia	23

Count in the last column is the number of species in OrthoDB v10.1 for the clade after excluding *Ornithorhynchus anatinus* from the mammalian clade

**Table 2:** Number of benchmark proteins in orthologous pairs recovered perfectly or as essentially complete by coding regions assembled by different methods

Read	Target	Ortho pairs		Perfect					Essentially complete				
set	species	Count	Median	rnaSP	Trinity	SPAln	Clust	SP10	rnaSP	Trinity	SPAln	Clust	SP10
Corn	Z. marina	2710	57.78	537	539	213	312	**548**	1434	**1450**	1037	916	1344
	A. tauschii	2888	77.94	550	555	410	510	**664**	1491	1514	1360	1395	**1552**
	S. bicolor	2871	92.60	549	554	463	572	**666**	1488	1507	1399	1464	**1549**
Thale	P. axillaris	2287	61.56	**1616**	0	598	1216	1598	1902	0	1364	1503	**1933**
cress	B. rapa	2312	85.18	1636	0	1255	1405	**1870**	1924	0	1879	1697	**2107**
	A. thaliana	2317	100.00	1640	0	1689	1375	**1951**	1928	0	1991	1668	**2122**
Worm	T. spiralis	2194	40.92	**1588**	0	156	646	1046	**1940**	0	638	867	1269
	C. briggsae	3112	86.46	2230	0	1216	2016	**2436**	2707	0	2336	2486	**2731**
	C. elegans	3130	100.00	2244	0	2276	2176	**2640**	2724	0	2720	2624	**2851**
Mouse	P. cinereus	9005	77.04	2824	2880	1954	2185	**3308**	3790	3743	3393	3202	**4274**
	H. sapiens	9109	86.73	2831	2889	2212	2568	**3469**	3803	3753	3537	3640	**4356**
	M. musculus	9177	100.00	2858	2914	2862	3001	**3686**	3832	3781	3850	3982	**4460**
Human	P. cinereus	8984	78.80	2796	3107	2144	2206	**3340**	4383	4645	3995	3551	**4792**
	M. Musculus	9109	86.73	2824	3134	2372	2401	**3496**	4425	4684	4168	3784	**4875**
	H. sapiens	9157	100.00	2839	3152	2994	2601	**3760**	4448	4708	4630	4032	**5013**

SAUTE_PROT low (SP10) with maximum of 10 variants reported per graph, SPAligner (SPAln), and CLUSTER (Clust) used proteins from the target species for assembling the read set. rnaSPAdes (rnaSP) and Trinity are de-novo assemblers. The median percent identity between orthologous protein pairs varies from 40.92 to 100%. In each row, count for the method that finds the largest number of proteins as perfect or as essentially complete are in bold

**Table 3:** *Drosophila melanogaster* and *Drosophila innubila* proteins in THO complex genes, their lengths, and alignment percent identity between orthologous pairs

		Drosophila melanogaster		Drosophila innubila		Percent
Gene	Isoform	Protein	Length (aa)	Protein	Length (aa)	Identity (%)
	A	NP_722763.1	1641			79.42
tho2	B	NP_608646.3	1642	XP_034472414.1	1660	79.38
	C	NP_001259905.1	1641			79.32
thoc5		NP_611856.1	616	XP_034478414.1	616	63.21
thoc6	All	NP_648557.1	350	XP_034481127.1	346	73.45
Hpr1		NP_649594.1	701	XP_034485608.1	716	77.59
	A	NP_728489.2	288			70.55
thoc7	B	NP_612011.1	287	XP_034481424.1	273	70.18

**Fig. 3:**
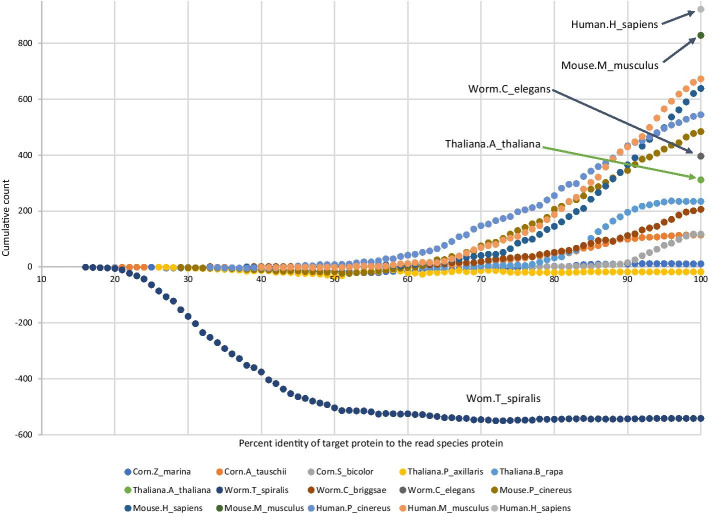
RNA-seq assembly comparison using BUSCO set: Number of additional proteins recovered perfectly by SAUTE_PROT low with a maximum of 10 variants reported per graph compared to rnaSPAdes is shown as a function of the percent identity of the alignment between the target and read protein. SAUTE_PROT low performs worse than rnaSPAdes only for the worm reads assembled using *Trichinella spiralis* target, but slightly outperforms rnaSPAdes for the small subset of *Trichinella spiralis* target sequences whose alignment to worm proteins have identity $\ge$ 75%

Figure 3 plots number of additional proteins recovered perfectly by SAUTE low with maximum of 10 variants reported per graph compared to rnaSPAdes as a function of the percent identity of the alignment between the target and read protein pairs for rows shown in Table 2. These results show that when percent identity of orthologous proteins used as target is at least 75%, SAUTE low even with a maximum of 10 variants reported per graph finds more coding regions that recover proteins perfectly or as essentially complete for all read and target set combinations.

### Assessment of RNA-seq set for THO genes

For RNA-seq THO set, we used eight *Drosophila melanogaster* proteins for five genes in the THO complex as benchmark. Table 3 shows proteins from *Drosophila melanogaster* and *Drosophila innubila* used as targets for SAUTE, CLUSTER, and SPAligner assemblies. Table 4 shows that SAUTE finds more coding regions that recover benchmark proteins perfectly compared to rnaSPAdes, Trinity, and CLUSTER. We did not include results for SPAligner assemblies in Table 4 as they are not an improvement over rnaSPAdes assemblies for this data set. SPAligner does not recover assembly for NP_608646.3 on SRR10541159 read set with either target protein. SPAligner assemblies using *Drosophila innubila* proteins as target sequences also have lower coverage or percent identity for an additional 11 assemblies compared to rnaSPAdes.

**Table 4:** Comparison of *Drosophila melanogaster* protein recovery for genes in THO complex

Gene and isoform	Method/Reads	SRR10541157	SRR10541159	SRR10541107	SRR10541200	SRR10541164
	Read bases (Gb)	2.51	2.78	2.95	3.47	4.49
tho2 isoform A	SAUTE w/ D. mel	1..1527, 100%	**Full, 100%**	**Full, 100%**	**Full, 100%**	**Full, 100%**
	SAUTE w/ D. inn	1..1527, 100%	**Full, 100%**	**Full, 100%**	**Full, 100%**	**Full, 100%**
	rnaSPAdes	Not found	Not found	Not found	Not found	Not found
	Trinity	Not found	**Full, 100%**	Not found	Not found	Not found
	Clust w/ D. mel	Not found	Not found	Not found	Not found	**Full, 100%**
	Clust w/ D. inn	Not found	Not found	Not found	Not found	Not found
tho2 isoform B	SAUTE w/ D. mel	**Full, 100%**	**Full, 100%**	**Full, 100%**	**Full, 100%**	**Full, 100%**
	SAUTE w/ D. inn	**Full, 100%**	**Full, 100%**	**Full, 100%**	**Full, 100%**	**Full, 100%**
	rnaSPAdes	**Full, 100%**	**Full, 100%**	**Full, 100%**	**Full, 100%**	**Full, 100%**
	Trinity	**Full, 100%**	1..1528, 100%	**Full, 100%**	**Full, 100%**	**Full, 100%**
	Clust w/ D. mel	**Full, 100%**	**Full, 100%**	**Full, 100%**	**Full, 100%**	Not found
	Clust w/ D. inn	Not found	Not found	Not found	Not found	Not found
tho2 isoform C	SAUTE w/ D. mel	Not found	Not found	Not found	Not found	1..1539, 100%
	SAUTE w/ D. inn	Not found	Not found	Not found	Not found	1..1539, 100%
	rnaSPAdes	Not found	Not found	Not found	Not found	Not found
	Trinity	Not found	Not found	Not found	Not found	Not found
	Clust w/ D. mel	Not found	Not found	Not found	Not found	Not found
	Clust w/ D. inn	Not found	Not found	Not found	Not found	Not found
thoc5	SAUTE w/ D. mel	**Full, 100%**	**Full, 100%**	Full, 100%	Full, 99.5%	**Full, 100%**
	SAUTE w/ D. inn	**Full, 100%**	**Full, 100%**	Full, 100%	Full, 99.5%	**Full, 100%**
	rnaSPAdes	Full, 99.8%	**Full, 100%**	Full, 100%	Full, 99.5%	Full, 99.7%
	Trinity	Full, 99.8%	**Full, 100%**	Full, 100%	Full, 99.5%	Full, 99.7%
	Clust w/ D. mel	**Full, 100%**	**Full, 100%**	Full, 100%	Full, 99.5%	Full, 99.7%
	Clust w/ D. inn	**Full, 100%**	Full, 99.8%	Full, 100%	Full, 99.5%	**Full, 100%**
thoc6	SAUTE w/ D. mel	Full, 99.7%	Full, 99.7%	**Full, 100%**	Full, 99.7%	Full, 99.7%
	SAUTE w/ D. inn	Full, 99.7%	Full, 99.7%	**Full, 100%**	Full, 99.7%	Full, 99.7%
	rnaSPAdes	Full, 99.7%	Full, 99.7%	Full, 99.7%	Full, 99.7%	Full, 99.7%
	Trinity	Full, 99.7%	Full, 99.7%	Full, 99.7%	Full, 99.7%	Full, 99.7%
	Clust w/ D. mel	Full, 99.7%	Full, 99.7%	Full, 99.7%	Full, 99.7%	Full, 99.7%
	Clust w/ D. inn	Full, 99.7%	Full, 99.7%	Full, 99.7%	Full, 99.7%	Full, 99.7%
Hpr 1	SAUTE w/ D. mel	Full, 100%	Full, 100%	Full, 100%	Full, 100%	Full, 100%
	SAUTE w/ D. inn	Full, 100%	Full, 100%	Full, 100%	Full, 100%	Full, 100%
	rnaSPAdes	Full, 100%	Full, 100%	Full, 100%	Full, 100%	Full, 100%
	Trinity	Full, 100%	Full, 100%	Full, 100%	Full, 100%	Full, 100%
	Clust w/ D. mel	Full, 100%	Full, 100%	Full, 100%	Full, 100%	Full, 100%
	Clust w/ D. inn	Full, 100%	Full, 100%	Full, 100%	Full, 100%	Full, 100%
thoc7 isoform A	SAUTE w/ D. mel	Not found	Not found	24..288, 100%	10..288, 100%	Not found
	SAUTE w/ D. inn	Not found	Not found	24..288, 100%	10..288, 100%	Not found
	rnaSPAdes	Not found	Not found	Full, 99.7%	10..288, 100%	Not found
	Trinity	Not found	Not found	Full, 99.7%	10..288, 100%	Not found
	Clust w/ D. mel	Not found	Not found	Full, 99.7%	10..288, 100%	Not found
	Clust w/ D. inn	Not found	Full, 99.7%	12..288, 99.6%	10..288, 100%	Not found
thoc7 isoform B	SAUTE w/ D. mel	Not found	Not found	Not found	10..287, 100%	Not found
	SAUTE w/ D. inn	Not found	Not found	Not found	Not found	Not found
	rnaSPAdes	Not found	Not found	Not found	Not found	Not found
	Trinity	Not found	Not found	Not found	Not found	Not found
	Clust w/ D. mel	Not found	Not found	Not found	Not found	Not found
	Clust w/ D. inn	Not found	Not found	Not found	Not found	Not found

SAUTE_PROT low (SAUTE) and CLUSTER (Clust) used proteins in Table 3 for *Drosophila melanogaster* (D. mel) and *Drosophila innubila* (D. inn) as targets. Cells in bold and highlighted in yellow show cases where the D. mel protein is recovered perfectly and there is at least one method that does not recover it perfectly. Proteins recovered as full length are marked as ’Full’; otherwise, coordinates on D. mel protein recovered are provided

**Table 5:** Sensitivity and precision achieved by different methods using AMRFinderPlus calls made on assemblies of 763 read sets and the corresponding finished assembly in FDA-ARGOS set

Set	True	False	False	Sensitivity	Precision
	positive	positive	negative		
SAUTE default	2801	575	22	0.99	0.83
SAUTE low	2803	833	20	0.99	0.77
SKESA	2674	308	149	0.95	0.90
SKESA + SAUTE default	2801	577	22	0.99	0.83
SKESA + SAUTE low	2803	834	20	0.99	0.77
SPAdes	2716	794	107	0.96	0.77
plasmidSPAdes	915	362	1908	0.32	0.72
SPAdes + plasmidSPAdes	2720	809	103	0.96	0.77
Cluster	2756	1209	67	0.98	0.70
SPAligner	2738	925	85	0.97	0.75

**Fig. 4:**
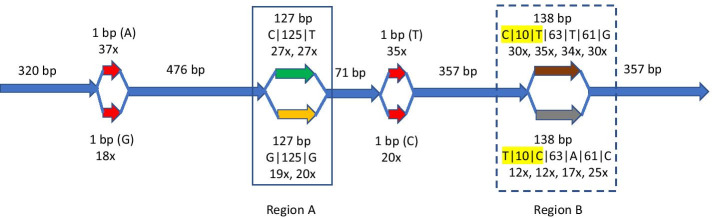
Variants reported in assembly of SRR10541157 by SAUTE low for *thoc5* protein. SAUTE produces correct variants using pairing information in reads for region A while the variant produced by both rnaSPAdes and Trinity is not supported by any paired read. Region B shows haplotyping achieved using reads alone as highlighted in yellow and additional haplotyping achieved using pairing information

Figure 4 highlights an example illustrated by assembly of SRR10541157 using target NP_611856.1 where SAUTE is able to correctly produce a variant using pairing information in reads while the variant produced by rnaSPAdes, Trinity, and SPAligner are not supported by any paired read. We note that assemblies produced by SAUTE using XP_034478414.1 as target that has only 63.2% identity to NP_611856.1 are same as that produced by using NP_611856.1 as target. It is interesting that CLUSTER with either target protein also produces the correct variant for this read set.

### Assessment of AMR set

FDA-ARGOS database [35] consists of regulatory grade sequences for microbes. It contains both the finished assembly and read sets for the same sample that makes it suitable to be used as a benchmark for comparing assemblies of read sets. AMRFinderPlus reports on the finished assembly and assemblies produced by different tools for the corresponding read sets were used to find gene calls made by an identical sequence match (tagged as ALLELEX or EXACTX by AMRFinderPlus) to assess if the expected variants were recovered by the assemblies produced by different methods.

Additional file 2 gives the read sets and corresponding finished assembly for AMR set. Table 5 shows the sensitivity and precision achieved by assemblies produced by SKESA, SPAdes, SAUTE, plasmidSPAdes, CLUSTER, and SPAligner when we use the calls on the corresponding finished assembly as the benchmark. It shows that SAUTE has higher sensitivity and precision compared to calls made on genome assemblies by SPAdes, calls made on genome assemblies by SPAdes supplemented by calls made on plasmidSPAdes assemblies, calls made on CLUSTER assemblies, and calls made on SPAligner assemblies. It also has better sensitivity compared to calls made on genome assemblies by SKESA but worse precision. SKESA has lower true positive and higher false negative count compared to SAUTE because the conservative heuristics in SKESA can cause some AMR genes that can be assembled by SAUTE to be only partially assembled or split in multiple contigs. SKESA also has lower false positive count compared to SAUTE as it has more aggressive filtering for carryover contamination.

**Table 6:** Number of variants produced by graphs generated by SAUTE default on AMR set and SAUTE_PROT low on BUSCO set

Number of	Number of graphs (percent %)	
variants	AMR	BUSCO
1	177,185 (96.83)	607,609 (60.16)
2	4407 (2.41)	204,994 (20.30)
3	230 (0.13)	34,205 (3.39)
4	946 (0.52)	63,283 (6.27)
5-10	172 (0.09)	53,370 (5.28)
11-100	50 (0.03)	41,143 (4.07)
101-1000	5 (0)	4316 (0.43)
$>\,1000$	0 (0)	1054 (0.10)
Total	182,995	1,009,974

**Table 7:** Species and number of read sets for the species assembled in the pathogen detection pipeline using SAUTE for antimicrobial resistance genes as of July 28, 2020

Species	Number of read sets
Salmonella enterica	278,133
E.coli and Shigella	89,600
Campylobacter jejuni	51,750
Listeria monocytogenes	32,124
Klebsiella pneumoniae	17,381
Enterococcus faecium	14,072
Neisseria	9308
Pseudomonas aeruginosa	4594
Vibrio cholerae	3556
Acinetobacter baumannii	3204
Enterococcus faecalis	3176
Legionella pneumophila	2848
Clostridioides difficile	1439
Enterobacter	1319
Staphylococcus pseudintermedius	1253
Vibrio parahaemolyticus	1170
Candida auris	744
Serratia marcescens	709
Mycobacterium tuberculosis	539
Citrobacter freundii	494
Klebsiella oxytoca	390
Vibrio vulnificus	365
Providencia alcalifaciens	253
Clostridium perfringens	223
Cronobacter	148
Corynebacterium striatum	98
Clostridium botulinum	95
Aeromonas hydrophila	26
Morganella morganii	20
Elizabethkingia anophelis	19
Kluyvera intermedia	1
Total	519,051

### Multiple variants in a graph

The distribution of number of variants produced for each graph in AMR and BUSCO sets when assembled using SAUTE default and SAUTE low, respectively, is shown in Table 6. Only one graph in sets for THO genes produced 32 variants, seven produced 16 variants, and remainder had a maximum of 8 variants. Table 6 shows that for most graphs, only a few variants are produced but some graphs can produce a large number of variants. Additional file 1 shows that a few more benchmark proteins can be recovered perfectly or as essentially complete when maximum of 100 or 1000 variants are reported per graph instead of 10. We suggest that for applications such as AMR gene detection and studying THO genes that are interested in finding existence of specific variants, default of a maximum of 1000 variants reported per graph is used while for applications such as annotation, a smaller number such as 10 is sufficient.

As an example, the maximum number of variants produced in any graph when mouse reads are assembled using mouse proteins is 103,356 with NP_631888.1 as target. Figure 2 shows that SAUTE low finds four de Bruijn subgraphs using NP_631888.1 as target. Variants produced recover seven known proteins for mouse perfectly, one reported variant has a retained intron, and 103,356 variants come from the subgraph that is composed of low-complexity sequences. Of the seven proteins recovered perfectly by SAUTE low, Trinity recovers three perfectly, rnaSPAdes recovers only one perfectly and one as essentially complete, SPAligner recovers one perfectly, and CLUSTER does not recover any proteins perfectly or as essentially complete. We note that using the option that collapses SNPs into ambiguous bases, SAUTE low reports 220 variants for the low-complexity subgraph instead of 103,356. This example shows that number of variants reported by SAUTE can be high for targets with low-complexity regions, but it also shows that even with such targets, it finds more correct variants not found by rnaSPAdes and Trinity.

### Production usage

As of July 28, 2020, SAUTE had been used by NCBI pathogen detection pipeline to assemble AMR genes for over 500,000 read sets including assemblies for *Salmonella enterica* (278,133 assemblies), *E.coli* and *Shigella* (89,600 assemblies), *Campylobacter jejuni* (51,750 assemblies), *Listeria monocytogenes* (32,124 assemblies), and *Klebsiella pneumoniae* (17,381 assemblies). All species assembled and number of read sets assembled is shown in Table 7. These species are tracked in the pathogen detection pipeline because of their importance in detecting pathogens in the food supply chain and in hospitals.

## Conclusions

Illumina sequencing technology continues to be the dominant technology at this time but it has short reads and short insert size that make de-novo assembly of repeated regions a challenging problem. SAUTE assembler is designed for assembling genomic and RNA-seq reads sequenced using Illumina that utilizes user specified genomic regions or genes of interest for guiding the de-novo assembly. The assembly approach finds subgraphs in the de Bruijn graph that correspond to the user specified sequences and compares the assemblies to those sequences to stay close to the specified regions. We showed that for RNA-seq data, target proteins can have only 75% identity and still be used to produce complete coding regions from the reads. Examples of heavily studied subtrees at this level of divergence in the Tree of Life are organisms as divergent as e.g. placental mammals or cereal grasses. As the tree of life is filled in with better quality sequences suitable to be used as targets, SAUTE will yield better results for more read sets.

Genomic sequence assembly using antimicrobial resistance and virulence genes as target, RNA-seq sequence assembly with proteins from THO complex genes, and comparison with modules of SPAdes was used to show that SAUTE complements de-novo assemblers for genes that are not assembled and when some of the variants for the gene are not assembled by the de-novo assemblers. Two coverage parameters in SAUTE can be changed to assemble read sets with low-coverage. Another flag can be set for collapsing SNPs into ambiguous bases. Additional comparisons with Trinity, SPAligner, and assembling subsets of read that align to use specified sequences show that SAUTE outperforms these methods.

Future work on SAUTE includes exploring additional ways of analyzing complex de Bruijn graphs. We showed an example where current process is inadequate: the possible number of variants exceeds over hundred thousand and are from low-complexity regions. Another direction for future work on SAUTE is to incorporate long read data or known transcripts from additional species to make informed choices in such extreme cases.

## Supplementary Information

**Additional file 1.** Description and results for BUSCO set.

**Additional file 2.** Description of AMR set.

## Data Availability

The SAUTE source code is available on GitHub at https://github.com/ncbi/skesa/releases [45] SAUTE source code is freely available to the public for use with exception of bundled third party code. The third-party code contained in SAUTE release is available under GNU GPLv3. See https://github.com/ncbi/skesa/blob/master/LICENSE for details. Most of the data generated or analyzed during this study is included in this published article and its additional files. Any additional datasets analyzed or produced during the current study are available from the corresponding author on reasonable request. Project name: SAUTE Source code: https://github.com/ncbi/skesa/releases Operating system: Linux Other requirements: BOOST License: Freely available to the public for use with ex-ception of bundled third party code. The third-party code contained in SAUTE release is available under GNU GPLv3. See https://github.com/ncbi/SKESA/blob/master/LICENSE for details. Any restrictions to use by non-academics: None

## Abbreviations

SRA: Sequence read archive
AMR: Antimicrobial resistance
BUSCO: Benchmarking universal single-copy orthologs
DBG: de Bruijn graph
GFA: Graphical fragment assembly
